# Low Diagnostic Accuracy of Transthoracic Ultrasound for the Assessment of Spontaneous Pneumothorax in the Emergency Setting: A Multicentric Study

**DOI:** 10.3390/jcm13164861

**Published:** 2024-08-17

**Authors:** Carla Maria Irene Quarato, Antonio Mirijello, Marialuisa Bocchino, Beatrice Feragalli, Donato Lacedonia, Gaetano Rea, Roberta Lieto, Michele Maggi, Anela Hoxhallari, Giulia Scioscia, Aldo Vicario, Giuseppe Pellegrino, Luca Pazienza, Rosanna Villani, Salvatore Bellanova, Pierluigi Bracciale, Stefano Notarangelo, Paride Morlino, Salvatore De Cosmo, Marco Sperandeo

**Affiliations:** 1 Department of Medical and Surgical Sciences, Institute of Respiratory Diseases, Policlinico Universitario “Riuniti” di Foggia, University of Foggia, 71122 Foggia, Italy; carlamariairene.quarato@gmail.com (C.M.I.Q.); donato.lacedonia@unifg.it (D.L.); dr.anelahoxhallari@gmail.com (A.H.); giulia.scioscia@unifg.it (G.S.); 2 Department of Internal Medicine, Fondazione IRCCS Casa Sollievo della Sofferenza, 71013 San Giovanni Rotondo, Italy; s.decosmo@operapadrepio.it; 3 Respiratory Medicine Unit, Department of Clinical Medicine and Surgery, Federico II University, 80131 Naples, Italy; marialuisa.bocchino@icloud.com (M.B.); al.vicario@studenti.unina.it (A.V.); 4 Department of Radiology, “SS. Annunziata” Hospital, University of Chieti, 66100 Chieti, Italy; beatriceferagalli@hormail.com; 5 Department of Radiology, Monaldi Hospital—Azienda Ospedaliera di Rilievo Nazionale (AORN) dei Colli, 80131 Naples, Italy; gaetano.rea71@gmail.com (G.R.); roblieto@gmail.com (R.L.); 6 Department of Emergency Medicine, Fondazione IRCCS Casa Sollievo della Sofferenza, 71013 San Giovanni Rotondo, Italy; milomaggi@alice.it (M.M.);; 7 Unit of Radiology, Fondazione IRCCS Casa Sollievo della Sofferenza, 71013 San Giovanni Rotondo, Italy; l.pazienza@operapadrepio.it; 8 Department of Medical and Surgical Sciences, Institute of Internal Medicine, University of Foggia, 71122 Foggia, Italy; rosanna.villani@unifg.it (R.V.); salvatorebellanova@libero.it (S.B.); 9 Pneumology and Respiratory Semi-intensive Care Unit, Ostuni Hospital, 72017 Ostuni, Italy; dott.bracciale@tiscali.it; 10 Respiratory Diseases and Respiratory Rehabilitation, “Teresa Masselli Mascia” Hospital, 71016 San Severo, Italy; stenotar@gmail.com (S.N.); paridemorlino@gmail.com (P.M.); 11 Unit of Interventional and Diagnostic Ultrasound of Internal Medicine, Fondazione IRCCS Casa Sollievo della Sofferenza, 71013 San Giovanni Rotondo, Italy

**Keywords:** pneumothorax, transthoracic ultrasound, lung sliding, false positive, false negative

## Abstract

**Background**: Pneumothorax (PNX) represents a common clinical condition in emergency departments (EDs), requiring prompt recognition and treatment. The role of transthoracic ultrasounds (TUSs) in the diagnosis of PNX is still debated. We aimed to prospectively evaluate the accuracy of TUSs in the detection of spontaneous PNX in EDs. **Methods**: A total of 637 consecutive adult patients who presented to the EDs of four Italian hospitals complaining of acutely onset chest pain and dyspnoea were included in the study. Exclusion criteria were previous traumatic events, cardiogenic causes of pain/dyspnoea and suspected tension PNX. The absence of “lung sliding” (B-mode) and the “bar-code” sign (M-mode) were considered indicative of PNX in a TUS. Accuracy, sensitivity, specificity, and positive and negative predictive values (PPVs, NPVs) were calculated using a chest CT scan as reference. **Results**: Spontaneous PNX occurred in 93 patients: of those, 83 (89.2%) were correctly identified by TUSs. However, 306 patients with suspected PNX at TUS were not confirmed by chest CTs. The diagnostic accuracy of both the absence of “lung sliding” and “bar-code” sign during TUS was 50.4% (95% CI: 46.4–54.3), sensitivity was 89.2% (95% CI: 81.1–94.7), specificity was 43.8% (95% CI: 39.5–48.0), the PPV was 21.3% (95% CI: 19.7–23.1) and the NPV was 96.0% (95% CI: 92.9–97.7). **Conclusions**: TUS showed high sensitivity but low specificity in the identification of PNX in EDs. Relying exclusively on TUSs results for patients’ management in ED settings is neither suitable nor recommendable. TUS examination can be useful to strengthen the clinical suspicion of PNX, but its results should be confirmed by a chest X-ray or CT scan.

## 1. Introduction

Pneumothorax (PNX) is the abnormal collection of air in the pleural space, causing partial or complete lung collapse [1]. It can be spontaneous and non-spontaneous. Spontaneous PNX can be further divided in primary, occurring in otherwise healthy young individuals without any apparent underlying lung disease, and secondary, when associated with underlying respiratory disorders. On the contrary, non-spontaneous PNX can be traumatic or iatrogenic [2]. In the latter case, PNX is an expected complication of invasive procedures (e.g., transthoracic/transbronchial needle biopsy, central venous line placement, positive pressure ventilation) [1,2].

PNX is a common clinical condition in emergency departments (EDs). More than half of PNX cases occur without any trauma [1]. Secondary spontaneous PNX shows higher morbidity and mortality compared to the primary spontaneous one because of a reduced cardiopulmonary reserve in patients with pre-existing lung disease [3]. At present, available management options range from clinical observation to aspiration or drainage to surgical intervention. Given that, in vulnerable patients, even a small PNX may be life-threatening, prompt diagnosis and management is pivotal [3].

Portable chest X-rays (CXRs) represent the first exam to identify PNX in the emergency setting because it is easily available, rapid, non-invasive and inexpensive. Moreover, it is possible to estimate the PNX size with good accuracy [4]. On the other hand, chest CT scans remain the gold standard in the detection of small PNXs and in size estimation, being also useful in the identification of associated pleuro-pulmonary alterations [5]. However, its routine use is limited by a patient’s clinical instability.

Recently, transthoracic ultrasound (TUS) has gained popularity for the assessment of critically ill patients in EDs [6]. The advantages of TUS over other imaging techniques include wide availability, ease of use, absence of radiation exposure and contained cost.

Main ultrasound (US) signs associated with PNX are the absence of so-called “lung sliding” in B-mode and the “stratosphere” or “bar-code” sign in M-mode [7]. Other TUS signs reported in cases of PNX include the absence of ring down artefacts (also called “B-lines”) and the detection of a “lung point” [8]. “Lung sliding” is a dynamic sign consisting of horizontal back-and-forth movement of the hyperechogenic pleural line during respiration. On the contrary, the “lung point” sign consists of the junction between the normal sliding lung and the absence of sliding due to PNX [9,10]. The presence of air in the pleural space prevents the view of respiratory sliding beneath the lung in B-mode examination, while the M-mode sand-like appearance is replaced by parallel lines, producing the “bar-code” sign [9,10,11].

However, the actual role of TUS in the diagnosis of PNX is still debated. Despite this, some authors consider TUS’s sensitivity higher than a chest X-ray and similar to that of chest a CT scan [12], according to other authors, TUS’s sensitivity was lower than a chest X-ray for the identification of PNX, even in cases requiring a chest tube [13]. Indeed, under optimal technical conditions (i.e., a patient able to maintain a sitting position), TUS is able to explore only about 70% of the pleural surface facing the chest wall. The remaining 30% is unexplorable due to the presence of skeletal structures (the rib cage) as well as the mediastinal area [9]. In addition, subcutaneous emphysema, muscular, chondral or pleural calcifications, scabs and sutures further reduce the explorable pleuro-parenchymal surface [14]. Moreover, the lack of “lung sliding” (and of a “bar-code” sign) can be also identified among conditions with reduced movement between visceral and parietal pleura (e.g., severe respiratory failure, hyper-inflated lungs, subpleural bullous disease, pulmonary contusion, prior thoracic surgery, pleural adherences, atelectasis, lobar consolidation and large parenchymal tumours or simple apnoea) [15]. Similarly, a “pseudo-lung point” can be found in cases of lung contusions, subpleural bubbles, severe interstitial lung diseases and pleural adherences [16].

With this background, the aim of the present multicentre study was to evaluate the diagnostic accuracy of bedside TUSs in detecting spontaneous PNX in subjects presenting to the ED complaining of the sudden onset of acute chest pain and dyspnoea. Accuracy, sensitivity, specificity, and positive and negative predictive values of TUS examination were calculated using a CT scan as the reference standard.

## 2. Materials and Methods

This multicentre study was conducted between January 2021 and October 2022. Consecutive patients with a sudden onset of acute chest pain and dyspnoea were recruited from the Italian EDs of the “Casa Sollievo della Sofferenza” Research Hospital in San Giovanni Rotondo, Italy, the “Monaldi” Hospital in Naples, Italy, the “Teresa Masselli Mascia” Hospital in San Severo, Italy, and the Hospital of Ostuni, Italy. The sample size was calculated assuming a prevalence rate of 10% for PNX among patients consecutively admitted to an Italian ED [17]. Thus, at a significant type I error rate of 5% and a 95% confidence interval (CI), an ideal sample size of 139 subjects was obtained for each centre. The study followed the amended Declaration of Helsinki; the ethical committee of the “Casa Sollievo della Sofferenza” Research Hospital (San Giovanni Rotondo, Italy) approved the protocol (TACE-CSS, n 106/2018).

A complete TUS examination was performed as an extension of a physical examination. After TUS, every patient underwent a CT scan within three hours, and this chest CT scan was regarded as the reference standard to confirm the diagnosis of PNX and to ascertain the presence of any concomitant pleuro-pulmonary conditions. In the case of clinical suspicion for cardiac disease or a known personal or familial history of ischaemic heart disease and/or diseases of the aorta, a preliminary cardiovascular evaluation with the cardiac injury markers 12-lead ECG and echocardiography was performed.

Inclusion criteria were as follows: (1) age over 18 years old, (2) CT scan performed within three hours of admission, (3) cardio-respiratory stability and (4) consent to participate in the study. Exclusion criteria were as follows: (1) known or suspected previous traumatic events, (2) recognised cardiogenic causes of pain/dyspnoea, (3) suspicion of tension pneumothorax (regarded as clinical or radiographic evidence of significantly increased intrapleural pressure causing haemodynamic compromise requiring urgent decompression) and (4) the denial or inability to give consent to participate in the study.

### 2.1. Ultrasound Examination

TUS examination was performed at each centre by an emergency physician with at least 10 years of experience. The employed ultrasound systems included the following: Mindray-7 (Mindray Medical Italy S.R.L., Trezzano sul Naviglio, Milan, Italy) or, alternatively, Esaote MyLab-30 (Esaote, Genoa, Italy) for the “Casa Sollievo della Sofferenza” Research Hospital in San Giovanni Rotondo; Samsung RS85 (Samsung Madison, Seoul, Republic of Korea) for the “Monaldi” Hospital in Naples; Esaote MyLab-40 (Esaote, Genoa, Italy) for the Hospital of San Severo and Esaote MyLab-7 (Esaote, Genoa, Italy) for the Hospital of Ostuni. All the ultrasound devices were equipped with a convex probe (3.5–8 MHz) and a linear transducer (8–14 MHz).

TUS was carried out using the correct setting for an adult thoracic study (gain: max 50%, focus pointed at the hyperechoic pleural line, activation of the tissue harmonic imaging) in B-mode and M-mode and employing the convex probe (3.5–8 MHz). Moreover, the linear probe (8–14 MHz) with vascular settings was also used for better definition of the pleural line. Patients were examined in a supine position. The examination included not only the evaluation on the anterior, lateral and posterior wall of chest surfaces but also the supraclavicular fossa and, finally, subxiphoid scans were performed to evaluate the heart (4 chambers) and the suprahepatic caval axis. These scans allowed for an assessment of the pericardium, cardiac cavities, their global kinetics and volaemia.

TUS examination was considered positive when it showed the absence of “lung sliding” in B-mode, followed by the presence of the “bar-code” sign in M-mode. The presence of a “lung point” was also assessed. Other TUS findings, such as the presence/absence of B-lines, presence/absence of pleural effusion, thickening (i.e., >3 mm with a convex probe; >2 mm with a linear probe) [18] and/or irregularity of the pleural line, were also evaluated.

TUS images were recorded and stored as dynamic videoclips and then blindly reviewed by a different operator with 35 years of experience. Cohen’s κ values in the interpretation of TUS results ranged from 0.80 to 1.00, indicating almost perfect agreement between operators. The mean time required to perform a TUS was approximately 10 min.

### 2.2. Statistical Analysis

Data are presented as means ± standard deviations (SD) for continuous variables and as numbers (n) and percentages (%) for descriptive variables. Accuracy, sensitivity, specificity, and positive and negative predicted values of the TUS examination in the identification of PNX were calculated with a 95% confidence interval (CI) using CT scans as a standard reference.

## 3. Results

**Figure 1:**
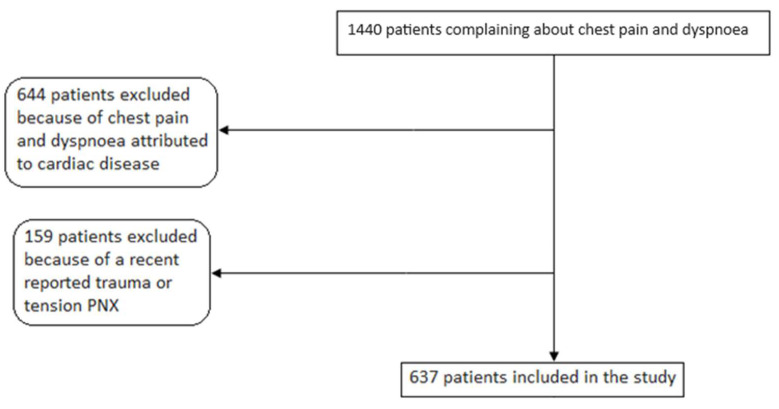
Study flow diagram.

From January 2021 to December 2022, 1440 consecutive patients complaining of sudden onset of dyspnoea with or without chest pain were evaluated. Among them, 644 patients were excluded because chest pain and dyspnoea were attributed to cardiac disease and 159 patients were excluded because of a recent reported trauma or tension PNX. Finally, 637 patients were included in the study, resulting in an acceptable sample size. The study flow diagram is depicted in Figure 1.

A total of 389 out of 637 (61.1%) patients received a diagnosis of PNX by TUS (positive results), while 248 examinations (38.9%) were judged negative. The gold standard (chest CT) diagnosis of PNX was made in 93 out of 637 (14.6%) patients, and it was excluded in 544 (85.4%) patients. When compared to the gold standard, TUS was able to correctly identify 83 out of 93 (89.2%) PNX cases (true positives) and to correctly exclude 238 of them (43.7%) (true negatives). Moreover, 306 cases of PNX identified by TUSs (78.6%) were not confirmed by a CT scan (false positives) and 10 cases of PNX not recognised by TUS (10.7%) were diagnosed by a chest CT (false negatives) (Table 1). Demographic and clinical characteristics of the study population are described in Supplementary Table S1.

When compared to a CT scan, the diagnostic accuracy of both the absence of “lung sliding” and the “bar-code” sign in a TUS was 50.4% (95% CI: 46.4–54.3) with a sensitivity of 89.2% (95% CI: 81.1–94.7), a specificity of 43.7% (95% CI: 39.5–48.0), a positive predictive value of 21.3% (95% CI: 19.7–23.1) and a negative predictive value of 96.0% (95% CI: 92.9–97.7). The diagnostic accuracy of the “lung point” in TUS was 54.3% (95% CI: 50.4–58.2) with a sensitivity of 89.3% (95% CI: 81.1–94.7), a specificity of 48.4% (95% CI: 44.1–52.6), a positive predictive value of 22.8% (95% CI: 21.0–24.8) and a negative predictive value of 96.3% (95% CI: 93.6–97.9).

As per the characteristics of PNX, a secondary spontaneous PNX was diagnosed in a total of 87/93 patients (93.5%) with known respiratory diseases. A right-sided pneumothorax was present in 49 cases (39 were small and 10 were large). A left-sided pneumothorax was diagnosed in 44 patients (35 were classified as small and 9 as large). As per echographic signs of PNX, the absence of “lung sliding” in a TUS’s B-mode (389 out of 637 patients, 61.1%) was always associated with the “bar-code” sign in M-mode. Absence of “lung sliding” and presence of the “bar-code” sign matched with the actual presence of PNX in a chest CT scan in 83 cases. Of them, 63 were small and 17 were large. Small PNX was diagnosed using a chest CT scan. A chest tube insertion was required in 52 cases; the other 31 patients improved with conservative treatment. True positive findings of PNX were detected in the anterior thoracic regions in 50/83 cases (60.2%), in the anterior-lateral thoracic regions in 25/83 cases (30.1%) and in the posterior-lateral thoracic regions in 8/83 cases (9.6%).

False positive results were obtained in 306/389 cases (78.7%). They were due to fibrothorax in 16/389 cases (4.1%), severe pulmonary fibrosis in 39/389 cases (10.0%), COPD exacerbation and subpleural bullae in 35/389 cases (9.0%), asthma exacerbation in 5/389 cases (1.3%), loculated effusion and empyema in 26/389 cases (6.7%), lung cancer in 46/389 cases (11.8%), pleural adhesions in 56/389 cases (14.4%), atelectasis in 34/389 cases (8.8%) and pneumonia in 40/389 cases (10.3%). False positive findings of PNX were also recorded in the upper anterior thoracic regions of 9/389 (2.3%) obese patients (BMI > 30 Kg/m^2^; mean: 31.5 ± 1.0 Kg/m^2^) whose symptoms were finally associated with other non-respiratory conditions.

False negative results were obtained in 10/93 cases of PNX. They were due to 2 small pneumothoraces restricted to the mediastinal area, 6 small apical pneumothoraces and 2 large posterior inferior pneumothoraces associated with pleural adhesion phenomena. Four patients required treatment with chest tube insertion.

The detection rate of the absence of “lung sliding”, the “bar-code” sign and the “lung point” in cases of PNX and other false positive conditions is detailed in Table 2.

Other less common TUS findings in PNX included the disappearance of subpleural masses or consolidation in the lung, a thickened and sometimes irregular pleural line in patients with pleural adhesions and chronic pulmonary comorbidities, and pleural effusion with the curtain sign at the air-fluid interface of a hydropneumothorax. In our 33 cases of hydropneumothorax, B-line artefacts have also been observed.

The detection rate of such less common TUS findings in cases of PNX and false positive conditions is detailed in Table 3.

## 4. Discussion

The present study shows that, in the emergency setting, the use of TUS examination for the diagnosis of PNX shows a low diagnostic accuracy as compared to a chest CT scan as a reference standard. In particular, TUS showed good sensitivity for the detection of PNX (89.3%, 95% CI: 81.1–94.7) but poor specificity both when considering “lung sliding” and “bar-code” signs (43.8%, 95% CI: 39.5–48.0) as diagnostic criteria, and when considering the “lung point” sign (48.4%, 95% CI: 44.1–52.6) as a diagnostic criterion.

Several meta-analyses have tried to investigate the accuracy of TUS in the diagnosis of PNX, mainly in trauma patients or subjects who had undergone percutaneous thoracic procedures, calculating a pooled TUS sensitivity of 78–98% and a pooled specificity of 85–99% [19,20,21]. In the present study, the enrolled population was highly heterogeneous and more reflective of a real-life setting considering that 527 out of 637 patients (82.7%) had pre-existing chronic respiratory diseases.

According to Lichtenstein and colleagues [22]—describing for the first time in the literature a practical alphabetical classification of lung ultrasound artefacts in the critically ill patient—the absence of “lung sliding” represents the most sensitive ultrasound marker for the diagnosis of PNX, with a sensitivity of 100% and a specificity of 78%, while the “lung point” is the most specific sign, with a sensitivity of 65% and a specificity of 100% [23]. However, it should be underlined that, in case of massive PNX with complete collapse of the lung to the hilum, the “lung point” is virtually absent. This diagnostic pitfall of the “lung point” should be known. The lower specificity of both “lung sliding” and “lung point” observed in our population could rely on the high prevalence of underlying chronic respiratory conditions affecting our sample and mimicking PNX in TUS [24]. These conditions were pleural adhesions (14.4%), lung cancer (11.9%), severe pulmonary fibrosis (10.0%), pneumonia (10.3%), COPD exacerbation and subpleural bullae (9.0%), atelectasis (8.8%), loculated effusion and empyema (6.7%), fibrothorax (4.1%), obesity (2.3%) and asthma exacerbation (1.3%).

It should be underlined that TUS can detect interstitial lung diseases (ILDs) if they involve the peripheral lung interstitium that is strictly adherent to the accessible superficial pleural surface. With this regard, severe fibrosis with honeycombing can be associated with the attenuation or abolition of the physiological gliding sign, aside from the irregularity or thickening of the pleural line and an increased number of B-lines [25].

**Figure 2:**
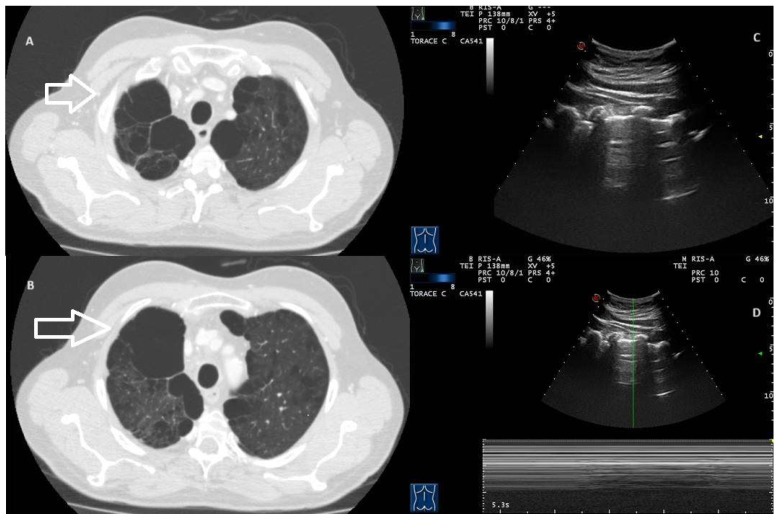
(**A**,**B**) Axial CT images showing extensive paraseptal emphysema characterised by voluminous thin-walled subpleural air bubbles in the right hemithorax (arrow). (**C**) Transthoracic ultrasound scan with a convex probe (7 MHz) corresponding to the area of paraseptal emphysema visualised on CT. Videoclip 1 shows the absence of a gliding sign. (**D**) Transthoracic ultrasound scan in M-mode showing the “bar-code” sign (absence of movement).

Even pathological conditions characterised by air trapping and lung hyperinflation (e.g., severe COPD and asthma exacerbations) may be associated with the absence of the physiological sliding sign, thus configuring a TUS “false positive” of PNX [14,25]. In case of subpleural bullae, the “lung sliding” may be minimal or absent because of the little movement of the visceral pleura that covers the bulla (Figure 2, Video S1).

Furthermore, a “lung point” may be visualised between the junction of normal lung tissue and a subpleural bulla [26,27]. As there is no free air in the pleural space, reverberation echoes producing B-line artefacts may be noticed in bullous disease [28].

**Figure 3:**
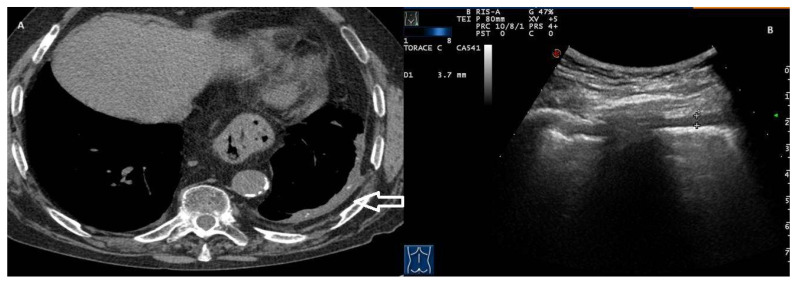
(**A**) Axial CT image with soft tissue window documents left, pleural thickening with calcifications and expansion of extrapleural fat (arrow); these features are suggestive of benign pleural fibrosis. Note the presence of a hiatal hernia. (**B**) Transthoracic ultrasound scan with a convex probe (7 MHz) corresponding to pleural fibrosis in the CT image, which shows an irregular hypoechogenic pleural thickening (3.7–5 mm). Videoclip 2 also shows the absence of a gliding sign with the presence of a “lung point”.

In our study, a thickened pleural line was observed in 7 out of 83 true cases of PNX (8.4%). A thickened pleural line associated with an attenuated or absent sliding sign represents a TUS finding associated with fibrothorax, a chronic sequela consisting of severe scarring and fusion of the pleural layers surrounding the lungs, leading to decreased movement of the lung and rib cage [9]. Although the thickening of the pleural line is very marked in fibrothorax cases, a thickened pleural line can also be observed in PNX, especially if it occurs in patients with adhesion phenomena and chronic pulmonary comorbidities (Figure 3, Video S2).

Furthermore, the absence of “lung sliding” was recorded in the upper anterior thoracic regions of 9 obese patients with other non-respiratory conditions. Indeed, obesity is an important cause of false positivity. Fat produces a decrease in the ultrasound speed as compared with soft tissue, leading to an “aberration” artefact consisting in the writing of structures behind fat distal to their actual position. This “aberration” may be misinterpreted as an immobile pleural line. Misjudgements often occur when scanning lung apexes due to the curvature of this area, the very tiny ultrasound window and the presence of three lung–pleural ligaments (namely, transversal–pleural, costal–pleural and vertebral–pleural), which limit view and movement [14].

According to some authors, most PNX false positives may be the underlying cause of dyspnoea in intensive care units [28]. On this issue, some authors replied, reporting that “lung sliding” has a negative predictive value of 99–100%, suggesting that the clear presence of such dynamic TUS findings effectively rules out a PNX condition [29]. On the other side, a massive PNX resulting in complete lung collapse will be, of course, detected as the absence of “lung sliding” in all evaluated chest regions. However, it is noteworthy to underline that TUS is able to explore only about the 70% of the pleural surface due to the obvious limitations represented by the air in the lung and the bony structures of the rib cage, thus missing possible PNX air pockets that may be trapped in different thoracic areas. As a result, examination limited to the anterior and lateral chest wall—as suggested by EFAST [30] and BLUE [31] protocols—may be not sufficient for a diagnosis of PNX.

**Figure 4:**
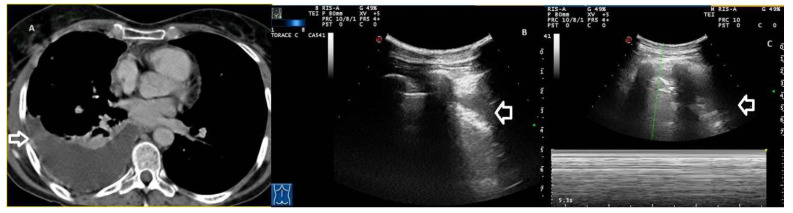
(**A**) Axial CT scan shows right pleural effusion with a slight thickening of the parietal pleura and an associated basal parenchymal hypoventilation suggestive of pleural empyema (arrow). (**B**) Transthoracic ultrasound scan with a convex probe (7 MHz) corresponding to the pleural empyema in the CT image, which shows a hyperechoic pleural effusion (arrow). Videoclip 3 also shows the absence of a gliding sign. (**C**) Transthoracic ultrasound scan in M-mode shows the “bar-code” sign (absence of movement) (arrow).

Despite our protocol requiring exploration of the entire thorax, we still missed the diagnosis in 10 cases of PNX. In 2 cases, TUSs failed to detect small PNXs restricted to the mediastinal area. Six false negative results were due to small PNXs located in the apical regions of the thorax, which is much less accessible for TUS due to the presence of the clavicles. Furthermore, TUS scans of the supraclavicular fossa were limited by the patients’ body habitus. In the remaining 2 cases, TUS was not able to identify 2 large posterior inferior areas of PNX. Possible explanations for these false negative results are the uneven distribution of PNX in the pleural cavity due to pleural adhesions and the difficulty in adequate TUS scanning of the posterior chest wall for symptomatic patients. Moreover, the increase in respiratory acts and contractions of the diaphragm in dyspnoeic patients may have led to a false perception of movement. On the contrary, TUS examination was able to assess all 33 posterior-lateral inferior cases (35.5%) of hydropneumothorax. Not surprisingly, TUS is a powerful imaging technique for the detection of pleural effusion, which is one of the main indications for its use. In patients with hydro-pneumothorax, TUS has revealed not only the absence of “lung sliding” but also the presence of the so-called “curtain” sign described by Targhetta in 1992 [32]. It consists of the projection of a free air “curtain”, associated with PNX, over the costophrenic recess, alternately obscuring the anechoic pleural effusion according to respiratory movement (Figure 4, Video S3).

Considering the high rate of false positive (306/544, 56.3%) and false negative (10/93, 10.8%) results, if the diagnostic workup would have been limited to TUS findings, a total of 306/637 subjects would have been erroneously diagnosed with PNX and scheduled for unnecessary, invasive treatment, while 10/637 patients would not have been properly diagnosed and adequately addressed. Indeed, only 4 of the 10 patients who finally received a diagnosis of PNX (false negatives from TUSs) required the insertion of a chest tube. However, the use of TUSs in EDs has complementary value by placing initial suspicion on PNX—which must be necessarily confirmed by other radiological examinations, such as a standard CXR and/or a CT scan—allowing for the detection of depth, not only extension as LUS does [14,33].

Due to the low specificity of TUS findings and the intrinsic limitations in the exploration of the pleuro-pulmonary surface, relying on TUS results to quickly make decisions on patient management in the ED setting should not be recommended.

A possible limitation of our study is that all the TUS examinations were performed by a single emergency physician per ED setting. However, all physicians were specifically trained and had at least 10 years of experience in TUS. Moreover, an independent review of TUS images by another physician with over 35 years of experience reduced potential bias due to inter-observer interpretation variability, even if inter-operator variability cannot be excluded. In fact, TUS is an operator-dependent imaging technique, and the evaluation of the pleural line’s movement mostly consists of a subjective overview, being heavily conditioned by the operator’s perception.

Furthermore, even intra-operator variability could be affected by probe position, as well as by patients’ body habitus, forced decubitus and respiratory rate. Thus, in our study, the absence of “lung sliding” showed a negative predictive value of 96%, but in other settings this value could be lower, implying a higher false negative rate depending on the patient’s characteristics, difficulty in performing the exam and the experience of the operator. Another possible limitation is that patients were enrolled from EDs of each study centre on a researcher-availability basis. However, as it was planned that at least one of the investigators would be available for most shifts and days throughout the study’s course, it is unlikely that this could have led to any selection bias in participants.

Finally, the LUS diagnosis of PNX was not based on the “lung pulse sign” [34]. The choice to consider only the absence of “lung sliding”, the “bar-code” sign and the “lung point” was based on the low specificity of the “lung pulse sign” for PNX. In fact, its presence is associated with any condition of dys-atelectasis (e.g., atelectasis, neoplastic and inflammatory lung consolidation, pneumonia, peri-bronchial fibrosis, lung fibrosis) [34], as a consequence, possibly leading to false positive results.

## 5. Conclusions

A fast ultrasound-guided evaluation at the bedside could represent a significant support to clinical evaluation and to formulate a diagnostic suspicion of PNX. However, TUS findings suggestive of PNX can also be observed in other acute and chronic pulmonary conditions, as well as in patients with obesity, a high respiratory rate, and forced decubitus, leading to false positive results [35].

In the present study, TUS showed a low diagnostic accuracy in the diagnosis of PNX, suggesting that a presumptive diagnosis of PNX should be confirmed by radiological examinations (e.g., chest X-ray and/or CT scan). Although chest CT scans remain the gold standard in the detection of PNX and in the identification of any underlying pulmonary conditions, in case of difficulty in performing a CT scan (e.g., due to the patient’s clinical instability), a bedside CXR remains a preferable option because, when a pneumothorax is suspected, further investigation is required to determine its depth.

## Figures and Tables

**Table 1:** Diagnostic accuracy of TUSs (“lung sliding”, “bar-code” sign, “lung point”) compared to the gold standard (chest CT scans) for the diagnosis of PNX.

	Chest CT Scan +	Chest CT Scan −	Total	
TUS +	83	306	389	PPV 21.3% (95% CI: 19.7–23.1)
TUS −	10	238	248	NPV 96.0% (95% CI: 92.9–97.7)
Total	93	544	637	
	sensitivity 89.2% (95% CI: 81.1–94.7)	specificity 43.7% (95% CI: 39.5–48.0)		diagnostic accuracy 50.4% (95% CI: 46.4–54.3)

Abbreviations: “+” : positive; “−“: negative.

**Table 2:** Detection rate of the absence of “lung sliding”, the “bar-code” sign and the “lung point” in cases of PNX and other false positive conditions.

Final Diagnosis	Absence of Lung Sliding	Bar-Code M-Mode	Lung Point
Pneumothorax	83/93	83/93	83/93
Fibrothorax	16/16	16/16	0/16
Pulmonary Fibrosis	39/87	39/87	39/87
Emphysema/Bullae	35/65	35/65	35/65
Asthma exacerbation	5/17	5/17	5/17
Loculated effusion/Empyema	26/26	26/26	26/26
Lung Cancer	46/100	46/100	46/100
Pleural adhesion	56/56	56/56	56/56
Atelectasis	34/46	34/46	34/46
Pneumonia	40/122	40/122	40/122
Obesity	9/9	9/9	0/9
Total	389/637	389/637	364/637

**Table 3:** Detection rates of less common TUS findings in cases of PNX and false positive conditions.

Final Diagnosis	Irregular Pleural Line	Thickened Pleural Line	Lesion	B-Lines	Pleural Effusion
Pneumothorax	6/83	7/83	0/83	33/83	33/83
Fibrothorax	12/16	16/16	0/16	8/16	1/16
Severe Fibrosis	25/39	38/39	1/39	37/39	1/39
Emphysema/Bullae	21/35	29/35	0/35	8/35	0/35
Asthma exacerbation	5/5	4/5	0/5	0/5	1/5
Loculated effusion/Empyema	10/26	11/26	0/26	24/26	26/26
Lung Cancer	10/46	10/46	42/46	30/46	11/46
Pleural adhesion	29/56	52/56	0/56	6/56	1/56
Atelectasis	3/34	7/34	1/34	31/34	33/34
Pneumonia	6/40	10/40	38/40	26/40	17/40
Obesity	0/9	0/9	0/9	0/9	0/9

## Data Availability

The data included in the present manuscript are available from the corresponding author upon reasonable request.

## Supplementary Materials

The following supporting information can be downloaded at: https://www.mdpi.com/article/10.3390/jcm13164861/s1.
