# Supplementary material for: Low Diagnostic Accuracy of Transthoracic Ultrasound for the Assessment of Spontaneous Pneumothorax in the Emergency Setting: A Multicentric Study
Source: J Clin Med. 2024 Aug 17;13(16):4861. doi: 10.3390/jcm13164861 (PMC11355464; doi:10.3390/jcm13164861)
Supplement: Supplementary file 1 [file jcm-13-04861-s001.zip › jcm-3107137-supplementary.pdf]

Supplementary Table S1. Demographic and clinical characteristics of the study population divided according to TUS results compared to final chest CT diagnosis (gold standard).

Characteristics	Total
Total number; n	637
Age; mean±SD	61.09±11.7
Sex, male; n (%)	418(65%)
BMI; mean±SD	26.83±1.9
Smokers; n (%)	177(44%)
CRD; n (%)	527(82.7%)
CCD; n (%)	457(71.7%)

Abbreviations: SD, Standard Deviation; BMI, Body Mass Index; CRD, Chronic Respiratory Diseases; CCR; Chronic Cardiovascular Diseases
